# Supplementary material for: Content-rich biological network constructed by mining PubMed abstracts
Source: BMC Bioinformatics. 2004 Oct 8;5:147. doi: 10.1186/1471-2105-5-147 (PMC528731; doi:10.1186/1471-2105-5-147)
Supplement: Additional File 5 — The original Chilibot query results of the term "long-term potentiation (LTP)" and 22 other terms, limiting the latest references analyzed to the years 1990, 1995, 2000, and 2004. [file 1471-2105-5-147-S5.bz2 › chilibotAdditionalFile5/ltp2000/html/PKA_CAMKII.html]

 


 **PKA** and **CAMKII** 
  
Found 39 abstracts in PubMed,  **30 abstracts were retrieved and analyzed**.  


---

 Search Google  |
 PDF files only 
|  EDU domain only 

---

**Interactive relationship** (e.g. stimulation, inhibition, etc)

- Previous studies have suggested that phosphorylation of Ser 16 by  **PKA**  is a prerequisite for Thr 17 phosphorylation by  **CaMKII**  and is essential to the relaxant effect of beta adrenergic stimulation.  Ref: 10825152 J Biol Chem, 2000
- Clearly, in CA1, both cascades are involved and are crucial, and the  **CaMKII**  cascade must precede the  **PKA**  cascade.  Ref: 9889075 Neurobiol Learn Mem, 1999
- Recent studies have shown that phosphorylation of AMPA receptors by cAMP dependent protein kinase  **PKA**  and calcium and calmodulin dependent protein kinase II  **CaMKII**  potentiates their activity, but phosphorylation of the receptor subunits may also affect their interaction with intracellular proteins, and their expression at the plasma membrane.  Ref: 11059799 Neurochem Res, 2000
- Serine 842 is phosphorylated by  **PKA** , PKC, and  **CaMKII**  in vitro and is phosphorylated in transfected cells by  **PKA** .  Ref: 10366608 J Neurosci, 1999

**Parallel relationship** (e.g. studied together, co-existance, homology, etc.)

- Taken together, these results suggest that MMA, at the same concentrations found in tissues of methylmalonic acidemic children, inhibits the in vitro activities of  **PKA** ,  **CaMKII**  and PP1 associated with the cytoskeletal fraction of the cerebral cortex of rats, a fact that may be involved with the pathogenesis of the neurological dysfunction characteristic of methylmalonic acidemia.  Ref: 9296563 Brain Res, 1997
- Based on the fact that this system contains cAMP dependent protein kinase  **PKA** , calcium calmodulin dependent protein kinase II  **CaMKII**  and protein phosphatase 1 PP1, we first tested the effect of MMA on the kinase activities by using the specific activators cAMP and calcium calmodulin or the inhibitors PKAI or KN 93 for  **PKA**  and  **CaMKII** , respectively.  Ref: 9296563 Brain Res, 1997
- Conversely, LTP induction in naive synapses and depressed synapses increases phosphorylation of the  **CaMKII**  site and the  **PKA**  site, respectively.  Ref: 10879537 Nature, 2000
- In this study, we analyzed in forebrain homogenates from female Sprague Dawley rats the activities of extracellular signal regulated kinase 1 2 ERK1 2, c Jun NH 2 terminal kinase JNK, glycogen synthase kinase 3beta GSK 3beta, cyclin dependent kinase 5 Cdk5, cAMP dependent protein kinase A  **PKA** , and calcium calmodulin dependent protein kinase II  **CaMKII**  at 0 n = n = n = 5, and 12 n = 5 h after heat shock and in non heat shocked controls n = 5.  Ref: 11121021 Proc Natl Acad Sci U S A, 2000
- We found that the phosphorylation at Ser 262 was several times more effective by  **CaMKII**  than  **PKA**  or PhK.  Ref: 9801171 FEBS Lett, 1998
- Moreover, co transfection of GluR6 receptor subunits with  **PKA**  alpha catalytic subunit or the calcium calmodulin dependent protein kinase II  [ **CAMKII** ]   **CamKII**  catalytic fragment did not change the sensitivity of the receptor to ethanol.  Ref: 9756045 Alcohol Clin Exp Res, 1998
- The amnesic effect of novelty appears to rely on N methyl D aspartate NMDA receptor and calcium calmodulin dependent protein kinase II  [ **CAMKII** ]   **CaMKII**  dependent... aspects of its habituation learning.  Ref: 10510197 Eur J Neurosci, 1999
- Ser 262 in human recombinant tau protein is a markedly more favorable site for phosphorylation by  **CaMKII**  than  **PKA**  or PhK.  Ref: 9801171 FEBS Lett, 1998
- PLB 24 possesses substrate properties as the native PLB as demonstrated by phosphorylation with exogenous, purified  **PKA** , cGMP dependent protein kinase PKG and a type II CaMK  **CaMKII** .  Ref: 9202842 Basic Res Cardiol, 1997
- Considering that the endogenous phosphorylating system associated with the cytoskeletal fraction contains cAMP dependent protein kinase  **PKA** , calcium calmodulin protein kinase II  **CaMKII** , and protein phosphatase 1 PP1, we first assayed the effect of the acid on the kinase activities by using the specific activators cAMP and calcium calmodulin or the inhibitors PKAI or KN 93 for  **PKA**  and  **CaMKII** , respectively.  Ref: 9344549 Exp Neurol, 1997
- The detection of spatial novelty is associated with a sequential activation of  **PKA** , ERKs p44 and p42 MAPKs and  **CaMKII**  and the phosphorylation of c AMP responsive element binding protein CREB in the hippocampus.  Ref: 11040265 Learn MemLearn Mem, 1997
- Treatment of Xenopus oocytes with specific inhibitors of PKC,  **PKA** ,  **CamKII** , tyrosine kinases, and serine threonine protein phosphatases did not affect the 100 mM ethanol induced inhibition of GluR6 receptor mediated currents.  Ref: 9756045 Alcohol Clin Exp Res, 1998
- In contrast, LTM appears to require  **PKA**  and PKC activity in both structures, guanylyl cyclase, PKG, and  **CaMKII**  activity in CA1, and MAPKK activity in the entorhinal cortex.  Ref: 10704324 Neurobiol Learn Mem, 2000
- Phosphorylation consensus sites for various kinases exist within the sequence of the potassium channel subunit Kv4.2, including consensus sites for extracellular signal regulated kinase mitogen activated protein kinase ERK MAPK, protein kinase A  **PKA** , protein kinase C PKC, and calcium calmodulin dependent kinase II  **CaMKII** , and kinase assays have shown that particular amino acids of the consensus sites are bonafide phosphorylation sites in vitro.  Ref: 11040264 Learn MemLearn Mem, 2000
- By deletion and point mutation analysis we show that phosphorylation by  **CaMKII**  and  **PKA**  occurs on a single serine residue at position 273.  Ref: 9677319 Biochem J, 1998
- Both cascades have been proposed to play a role in memory  **CaMKII**  in the early phase and  **PKA**  in the transition between the early phase and long term memory.  Ref: 9889075 Neurobiol Learn Mem, 1999
- The drugs and doses were the guanylyl cyclase inhibitor LY83583 LY, 2.5 microMg, the inhibitor of Tyr protein kinase at low concentrations and of protein kinase G PKG at higher concentrations lavendustin A LAV, 0.1 and 0.5 microMg, the PKG inhibitor KT5823 2.0 microMg, the protein kinase C PKC inhibitor staurosporin STAU, 2.5 microMg, the inhibitor of calcium calmodulin protein kinase II  **CaMKII**  KN62 3.6 microMg, the protein kinase A  **PKA**  inhibitor KT5720 0.5 microMg, and the mitogen activated protein kinase kinase MAPKK inhibitor PD098059 PD, 0.05 microMg.  Ref: 10704324 Neurobiol Learn Mem, 2000
- It has been established that the GluR1 subunit is phosphorylated on two distinct sites, Ser 831 and Ser 845, by  **CaMKII**  and by  **PKA** , respectively,.  Ref: 10901266 Cell Mol Neurobiol, 2000
- In contrast, in the amygdala, only the  **CaMKII**  cascade is active, and it does not play a central role in memory, inasmuch as its deleterious effect may be fully recovered by stimulation of the  **PKA**  cascade in the hippocampus.  Ref: 9889075 Neurobiol Learn Mem, 1999
- Both Ser 16 and Thr 17 of phospholamban PLB are phosphorylated, respectively, by cAMP dependent protein kinase  **PKA**  and calcium calmodulin dependent protein kinase II  **CaMKII** .  Ref: 10825152 J Biol Chem, 2000
- At 3 6 h, kinase assays specific for  **PKA**  and  **CaMKII**  showed no increased activity toward either tau or selective substrates.  Ref: 11121021 Proc Natl Acad Sci U S A, 2000
- To illustrate the capabilities of the technique, the activation of protein kinase C PKC, protein kinase A  **PKA** , calcium calmodulin activated kinase II  **CamKII** , and cdc2 protein kinase cdc2K was measured in response to both pharmacological or physiological stimuli.  Ref: 10700147 Nat Biotechnol, 2000
- Using phosphorylation site specific antibodies, we found that induction of chem LTD produces a persistent dephosphorylation of the GluR1 subunit of AMPA receptors at serine 845, a cAMP dependent protein kinase  **PKA**  substrate... a substrate of protein kinase C PKC and calcium calmodulin dependent protein kinase II  [ **CAMKII** ]   **CaMKII** .  Ref: 9856470 Neuron, 1998
- The phosphorylation of the glutamate receptor 1 GluR1 subunit of AMPA receptors by protein kinase A  **PKA** , protein kinase C PKC, and calcium calmodulin dependent protein kinase II  **CaMKII**  has been characterized extensively.  Ref: 10366608 J Neurosci, 1999
- Several studies showed that cAMP and type II calcium calmodulin dependent protein kinases  **PKA**  and  **CaMKII**  are markedly affected by typical AD in two different and complementary cellular districts, respectively microtubules a somatodendritic compartment and synaptic vesicles a presynaptic terminal compartment.  Ref: 9833637 Neuropharmacology, 1998
- Although they may contribute to basal NET expression, we could not implicate  **CaMKII** ,  **PKA**  or nitric oxide linked pathways in MCh regulation.  Ref: 9808704 J Pharmacol Exp Ther, 1998
- Ten minutes prior to the retention test, through indwelling cannulae placed in the CA1 region of the dorsal hippocampus, they received 0.5 microl infusions of saline, a vehicle 2% dimethylsulfoxide in saline, the glutamate NMDA receptor blocker, aminophosphonopentanoic acid AP5 5.0 microg, the AMPA kainate receptor blocker, cyanonitroquinoxaline dione CNQX 0.25 or 1.25 microg, the metabotropic receptor antagonist, methylcarboxyphenylglycine MCPG 0.5 or 2.5 microg, the inhibitor of calcium calmodulin dependent protein kinase II  [ **CAMKII** ]  KN62 3.5 microg, the inhibitor of cAMP dependent protein kinase  **PKA** , Rp cAMPs 0.1 or 0.5 microg, the stimulant of the same enzyme, Sp cAMPs 0.1 or 0.5 microg, or the inhibitor of the mitogen activated protein kinase MAPK kinase, PD098059 10 or 50 microM.  Ref: 10840135 Behav Brain Res, 2000
- In contrast, the protein synthesis inhibitor, cycloheximide, the selective inhibitor of cAMP dependent protein kinase  **PKA** , H 89, the selective inhibitors of protein kinase C PKC, PKCI and GF109203X, the mitogen activated protein MAP kinase kinase inhibitor, PD98059, or the inactive  **CaMKII**  inhibitors, KN 92 and NP218, had no effect on the currents.  Ref: 10101246 Brain Res Mol Brain Res, 1999
- The findings bear on the participation of  **CaMKII**  and of the cAMP  **PKA**  cascade in memory processes in the hippocampus and the amygdala.  Ref: 9889075 Neurobiol Learn Mem, 1999
- Taken together, these results demonstrate that PA inhibits the in vitro activities of  **PKA** ,  **CaMKII** , and PP1 associated with the cytoskeletal fraction of the cerebral cortex of rats.  Ref: 9344549 Exp Neurol, 1997
- LTP is differentially sensitive to  **CaMKII**  and  **PKA**  inhibitors depending on the history of the synapse.  Ref: 10879537 Nature, 2000
- The effects of behavioral tasks on the in vitro phosphorylation of intermediate filament subunits of rat hippocampus are mediated by  **CaMKII**  and  **PKA** .  Ref: 9138727 Brain Res, 1997
- In this study we compared the phosphorylation of tau at Ser 262 by  **CaMKII** , PhK and  **PKA**  in vitro as determined by radioimmunoblots developed by the monoclonal antibody 12E8 which recognizes P Ser 262 and P Ser 356.  Ref: 9801171 FEBS Lett, 1998
